# Photoporation of Biomolecules into Single Cells in Living Vertebrate Embryos Induced by a Femtosecond Laser Amplifier

**DOI:** 10.1371/journal.pone.0027677

**Published:** 2011-11-16

**Authors:** Yoichiroh Hosokawa, Haruki Ochi, Takanori Iino, Akihiro Hiraoka, Mikiko Tanaka

**Affiliations:** 1 Graduate School of Materials Science, Nara Institute of Science and Technology, Ikoma, Nara, Japan; 2 Graduate School of Biological Sciences, Nara Institute of Science and Technology, Ikoma, Nara, Japan; 3 Graduate School of Bioscience and Biotechnology, Tokyo Institute of Technology, Nagatsuta-cho, Midori-ku, Yokohama, Japan; Laboratoire Arago, France

## Abstract

Introduction of biomolecules into cells in living animals is one of the most important techniques in molecular and developmental biology research, and has potentially broad biomedical implications. Here we report that biomolecules can be introduced into single cells in living vertebrate embryos by photoporation using a femtosecond laser amplifier with a high pulse energy and a low repetition rate. First, we confirmed the efficiency of this photoporation technique by introducing dextran, morpholino oligonucleotides, or DNA plasmids into targeted single cells of zebrafish, chick, shark, and mouse embryos. Second, we demonstrated that femtosecond laser irradiation efficiently delivered DNA plasmids into single neurons of chick embryos. Finally, we successfully manipulated the fate of single neurons in zebrafish embryos by delivering mRNA. Our observations suggest that photoporation using a femtosecond laser with a high pulse energy and low repetition rate offers a novel way to manipulate the function(s) of individual cells in a wide range of vertebrate embryos by introduction of selected biomolecules.

## Introduction

Manipulation of gene function *in vivo* is an indispensable technique for modifying individual functions of targeted cells in living animals. Gene manipulation techniques have been applied for molecular and developmental biology as well as for gene therapy. Targeted gene manipulation can be conducted by producing transgenic animals expressing a transgene under the control of a specific promoter, but this method is time consuming and is only applicable for a limited range of organisms. For non-transgenic animals, a variety of vectors, chemicals, and electroporation-based procedures have been used for delivery of exogenous genes. Viral vectors have proven to efficiently deliver exogenous genes to cells of living animals; however, the virus or its vector derivatives may be toxic to the host animal. Liposome-mediated transfection (lipofection) and electroporation have also been widely used to deliver genetic materials; however, these methods come with the challenge of controlling the range of the transfected area. Recently, transgene expression in a transgenic worm was successfully manipulated by using a heat shock promoter and then focusing a continuous-wave infrared laser on a single cell in the transgenic animal [1]. Unfortunately, this system is only applicable to transgenic animals carrying a gene driven by the heat shock promoter.

Near-infrared femtosecond (NIR-fs) laser photoporation is an attractive method for delivering DNA plasmids into targeted cells. Recent work has demonstrated the successful application of this method to targeted cells in culture [2], [3], [4]. Most groups have utilized a NIR-fs laser oscillator with a low pulse energy (<1 nJ/pulse) and a high repetition rate (>10 MHz) for DNA delivery. It is, however, difficult to achieve single-cell manipulation in thick late-stage vertebrate embryos with low transparency using this approach (Figure S1A). Although an objective with a high numerical aperture and a short working distance is typically required for effective multiphoton absorption for transfection, an objective with a long working distance is required to focus through thick vertebrate embryos (>300 µm; Figure S1B). Because the numerical aperture becomes small and the laser focal spot size (diameter *d*) becomes large with increasing working distance, an intense NIR-fs laser pulse is required to obtain sufficient multiphoton absorption at the laser focal spot. However, NIR-fs laser pulses with a high pulse energy and high repetition rate are problematic as they lead to overheating due to non-radiation relaxation of photoexcitation energy at the laser focal spot. To avoid such overheating, the pulse repetition rate must be decreased to suppress total irradiation energy. Therefore, we have found that a NIR-fs laser with a high pulse energy and low repetition rate yields suitable laser irradiation conditions for living vertebrate embryos (Figure S1B).

**Figure 1:**
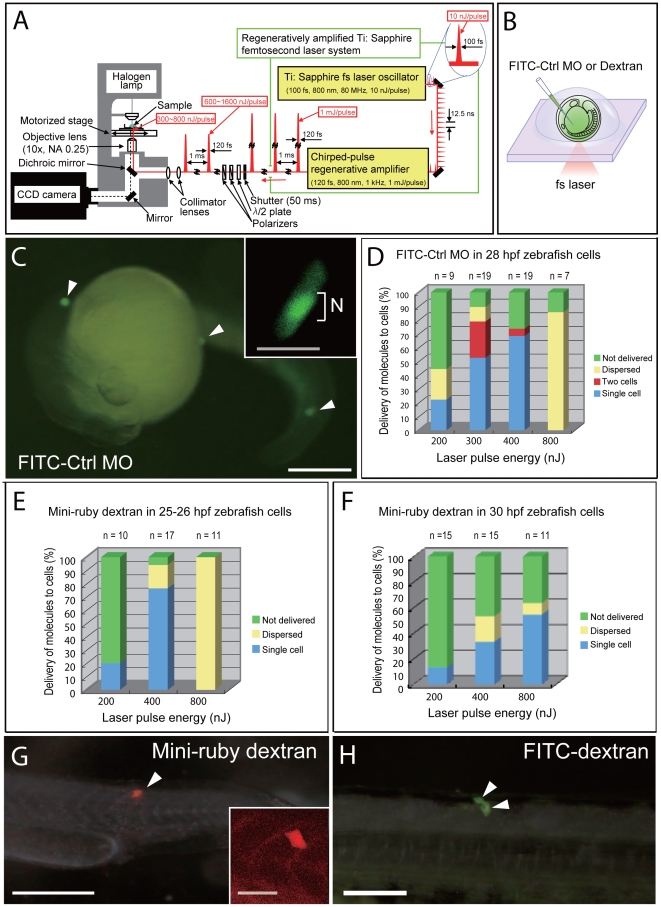
NIR-fs Laser Photoporation of MOs or dextran into single cells of zebrafish embryos. (A) Experimental setup for targeted introduction of biomolecules with femtosecond laser irradiation. Specifications are described in Materials and Methods. (B) Schematic representation of targeted delivery to single cells of zebrafish embryos. FITC-MO, FITC-dextran, or mini-ruby dextran was injected into the chorion cavity of anesthetized embryos mounted in methylcellulose solution. The femtosecond laser pulse train was focused on the surface of single cells. (C) FITC-MO was detected in three targeted single cells (arrowheads). Inset: FITC-MO was localized to the nucleus (N) of the targeted cell. (D) Success rate of delivery of FITC-MO into 28-hpf zebrafish epithelial cells. (E, F) Success rate of delivery of mini-ruby dextran into 25- to 26-hpf (E) and 30-hpf (F) zebrafish epithelial cells. (G) Mini-ruby dextran was detected in a single targeted cell (arrowhead) of a 25- to 26-hpf zebrafish embryo. Inset: Magnification of the targeted cell. (H) At 24 h after introduction of FITC-dextran, FITC fluorescence was detected in newly divided cells (arrowheads). n, number of individual experiments. Scale bars: 250 µm (C, G); 50 µm (insets in C, G); 100 µm (H).

Here, we demonstrate for the first time the effective introduction of dextran, DNA plasmids, antisense morpholino oligonucleotides (MOs), or mRNAs into targeted single cells in embryos of non-transgenic zebrafish, shark, chick, and mouse by photoporation using a femtosecond laser amplifier with a high pulse energy (>100 nJ) and a low repetition rate (1 kHz) [4] (Figure 1A and Figure S1B). We show that this method allowed us to manipulate the fate of individual neurons in non-transgenic zebrafish embryos by introducing mRNA in a targeted fashion.

## Results

First, we introduced FITC-tagged standard control morpholinos (FITC-MOs; 1,810 Da; Gene Tools; www.gene-tools.com) into single cells in living zebrafish embryos (Figures 1B–D). MOs are useful research tools for reverse genetics because they block targeted RNA sequences. FITC-MOs (5 ng/nl) were injected into the chorion cavity of anesthetized zebrafish embryos at 28 h post-fertilization (hpf), and the embryos were mounted in 3% methylcellulose in the depression of a glass slide (Figure 1B). NIR-fs laser pulses (120 fs, 800 nm, 50 pulses at 1 kHz) were focused on targeted epithelial cells of these embryos through a 10× objective on an inverted microscope (Figure 1A–B). Immediately after irradiation, delivery of MOs was observed under a fluorescence microscope. When laser pulses with an energy of 200 nJ/pulse were used, FITC fluorescence was observed in single cells in 22% of cases (2 single cells out of 9 individual shots; Figure 1D). When energies of 300 and 400 nJ/pulse were used, fluorescence was seen in single cells in 52.6% and 68.4% of cases (10/19 and 13/19, respectively; Figure 1D). Irradiation at 800 nJ/pulse led to physical dispersion of cells around the targeted area in 85.7% of cases (6/7; Figure 1D). Thus, a laser pulse with an energy of 300–400 nJ/pulse provided the efficient delivery of FITC-MOs into single cells of 28 hpf zebrafish embryos (Figure 1C–D).

Next, we delivered a relatively larger molecule, 10,000-Da dextran, into single cells of 25- to 26-hpf zebrafish embryos (Figure 1E, G). We injected dextran into the chorion cavity, irradiated embryos, and observed fluorescence of dextran immediately after irradiation. Irradiation with 400-nJ laser pulses provided the best conditions for delivering dextran to single cells, with a success rate of 76.5% (13/17; Figure 1E). With 800-nJ laser pulses, cells were dispersed in all cases (11/11; Figure 1E). At 24 h post-irradiation with a 400-nJ/pulse, dextran fluorescence was detected in newly divided cells (Figure 1H), suggesting that irradiated cells remained healthy. When dextran was introduced into single cells of 30-hpf embryos, laser pulses at 800 nJ/pulse delivered the dextran into individual cells in 54.5% of cases (6/11; Figure 1F).

Taken together, these observations suggest that our photoporation technique can be used to introduce molecules with a molecular size of up to 10,000 Da into single cells in zebrafish embryos and that higher pulse energy is required to introduce molecules into the cells of later-stage embryos. Furthermore, higher energies from the NIR-fs laser tend to physically disperse cells in early-stage embryos.

**Figure 2:**
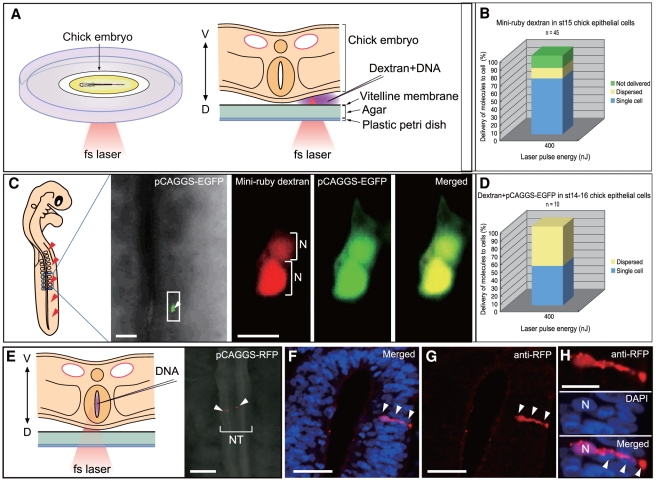
NIR-fs Laser Photoporation of dextran or DNA into single cells of chick embryos. (A) Schematic representation of targeted delivery to single cells of chick embryos. Mini-ruby dextran and pCAGGS-EGFP were injected between the vitelline membrane and the embryo in the New culture system, and the femtosecond laser was focused on targeted cells. (B) Success rate of delivery of mini-ruby dextran into stage 15 chick epithelial cells. (C) At 15 h after introduction, the fluorescence of both mini-ruby dextran (red) and EGFP (green) transcribed from the plasmid vector was detected in an actively dividing cell (white arrowhead). (D) Success rate of delivery of mini-ruby dextran and pCAGGS-EGFP into stage 14–16 chick epithelial cells. (E–G) pCAGGS-RFP was transfected into single targeted cells in chick embryos. DNA was injected into the lumen of the neural tube, and the femtosecond laser was focused on individual cells. (E) At 15 h after introduction, dorsal view shows RFP labeling of targeted neurons (arrowheads). (F–H) At 15 h, transverse sections were visualized with DAPI (blue) and RFP antibody (red). (F) Merged image of anti-RFP (G) and DAPI. RFP was identified in a single neuron and its axon in the neural tube (arrowheads). (H) Higher magnification of transfected neuron in F and G. The nucleus (N) was identified by DAPI. D, dorsal; N, nucleus; NT, neural tube; V, ventral; n, numbers of individual experiments. Scale bars: 50 µm (left in C; E, F, G); 10 µm (right in C); 20 µm (H).

Next, we applied our photoporation technique to amniote chick embryos (Figure 2). Chick embryos were cultured using a modified version of the New method [5]. Briefly, stage 15 chick embryos attached to a paper ring were placed on an albumin agar plate, and dextran (10,000 Da) was injected between the vitelline membrane and the ectoderm (Figure 2A). Laser pulses of 400 nJ were focused on embryos from the dorsal side. Immediately after irradiation, we observed successful transfer of dextran into single epithelial cells in 71% of cases (32/45; Figure 2B).

Introduction of DNA plasmids is a general method for manipulating gene function in model organisms. To date, however, no studies have demonstrated successful manipulation of gene function in single cells of living amniote embryos. Thus, we introduced DNA into single cells in chick embryos. We injected a cocktail of mini-ruby dextran and a DNA plasmid (pCAGGS-EGFP) between the vitelline membrane and the epithelium of stage 14–16 chick embryos and irradiated single cells with 400-nJ laser pulses. At 15 to 24 h post-injection, 50% of cells showed fluorescence from mini-ruby dextran as well as EGFP (produced by the plasmid) in single cells or in newly dividing cells (Figure 2C, D).

**Figure 3:**
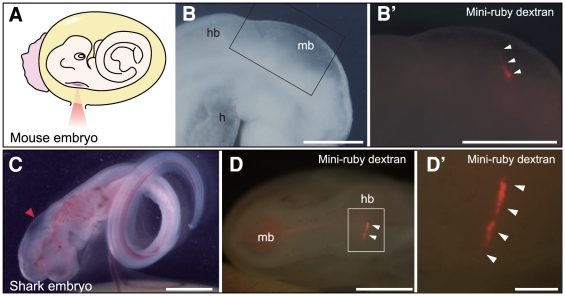
Targeted introduction of biomolecules into single neurons of mouse and shark embryos. Mini-ruby dextran was injected into the neural tube of mouse (A, B) and shark (C, D) embryos, and the femtosecond laser was used to irradiate targeted single neurons. (A) Schematic of targeted delivery to single neurons of E9 mouse embryos. (B) Lateral view of the head region in bright field. (B′) Higher magnification of the midbrain region shown in B. Dextran was identified in a single neuron (white arrowheads). (C) Side view of a cultured stage 29 shark embryo prior to the photoporation. Red arrowhead indicates the spot where the laser was focused. (D) Dorsal view of the head region. Dextran was identified in a single neuron (white arrowheads). (D′) Higher magnification of the hindbrain region shown in D. h, heart; hb, hindbrain; mb, midbrain. Scale bars: 200 µm (B, B′, D′); 2 mm (C); 1 mm (D).

To assess delivery to internal cells of chick embryos, we next introduced DNA into single neurons (Figure 2E–H). The pCAGGS-RFP construct was injected into the lumen of the neural tube in stage 12–14 chick embryos using a glass capillary needle and irradiated with 400-nJ laser pulses. We can target areas deep in the neural tube only by shifting the laser focal position in the *z* axis as shown in Movie S2. At 24 h post-injection, red fluorescent protein (RFP) was distributed in single neurons (Figure 2E). Immunohistochemical analysis confirmed that RFP was produced in the transfected neurons (Figure 2F–H). Thus, DNA constructs were successfully introduced to both external and internal cells using our photoporation technique. We then examined the usefulness of our photoporation technique in a range of vertebrate embryos (Figure 3) by introducing the larger molecule (10,000-Da dextran) into single neurons of embryos from a model animal (mouse) and a non-model animal (shark). E9 mouse embryos with closed yolk sacs were cultured as described [19], [20]. Laser pulses of 400 nJ were focused on the surface of the neural tube through a small slit made in the yolk sac (Fig. 3A). At 24 h post-injection, dextran was successfully delivered to single neurons of cultured mouse embryos (Fig. 3B). Stage 29 shark embryos were removed from the eggs (Fig. 3C) and transferred to a transparent dish filled with saline solution. Immediately after irradiation with 400-nJ laser pulses, shark embryos were returned to the eggs and cultured. At 3 days post-irradiation, dextran fluorescence was detected in single neurons of cultured shark embryos (Fig. 3D). From the four animals tested, it stands to reason that our photoporation technique could be applied to a wide range of vertebrate embryos.

**Figure 4:**
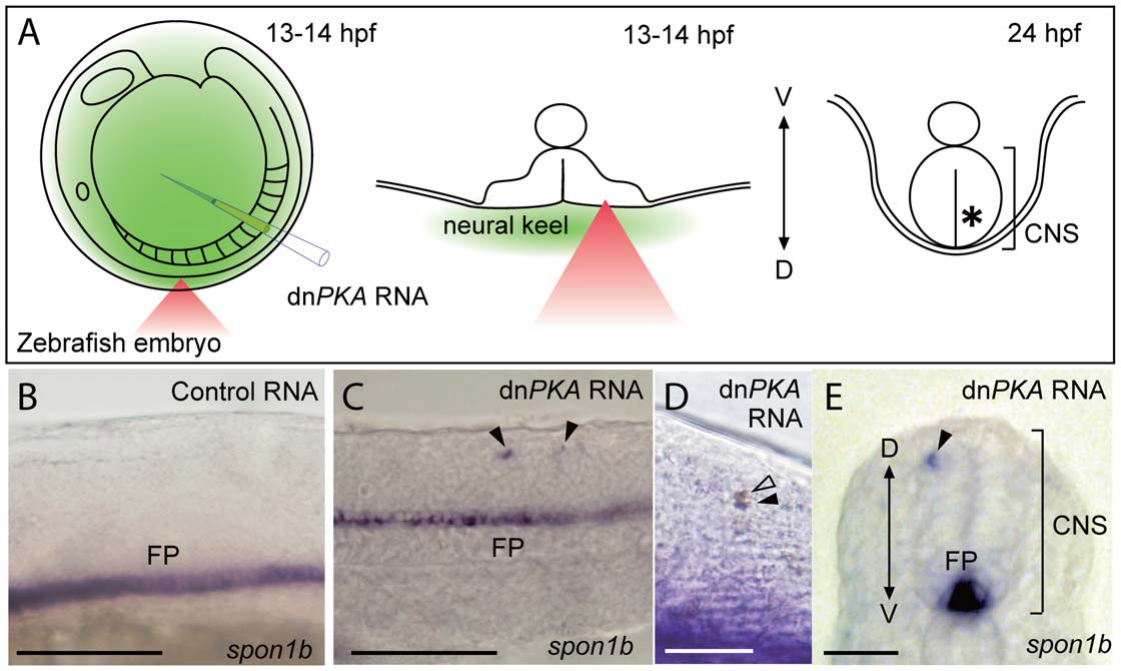
Manipulation of the fate of targeted cells in zebrafish embryos. Targeted introduction of dn*PKA* mRNA induced floor plate marker gene expression in single dorsal neurons of zebrafish embryos. (A) Schematic illustration of dn*PKA* mRNA delivery to single dorsal neurons of zebrafish embryos. dn*PKA* mRNA was injected into the cavity between the chorion and the surface of embryo, and dorsal neurons in the neural keel (center) were irradiated at 13–14 hpf with a femtosecond laser pulse (red triangle). Irradiated embryos were fixed at 24 hpf (right panel) and processed for *in situ* hybridization. (B–E) Expression of the floor plate marker *spon1b* in embryos injected with control *EGFP* RNA (B) and dn*PKA* RNA (C–E) at 24 hpf. (B–D) Lateral view; (E) transverse section. In the control embryo (B), *spon1b* transcripts were detected only in the floor plate. In the dn*PKA* RNA-injected embryo, ectopic *spon1b* signals were observed in individual cells (arrowheads in C, E). (D) Expression of the floor plate marker *spon1b* (purple) and localization of biotin-dextran (brown) in embryos injected with dn*PKA* RNA at 24 hpf. Ectopic expression of *spon1b* was only detected in cells photoporated with biotin-dextran (black arrowhead). Although dextran was also observed in the adjacent dividing cell (open arrowhead), ectopic *spon1b* expression was barely detected in this cell. CNS, central nervous system; D, dorsal; FP, floor plate; V, ventral. Scale bars: 100 µm (B, C); 50 µm (D, E).

In developmental biology, introduction of mRNA into single-cell embryos of teleost fishes is a useful method for gain-of-function experiments, and introduction of MOs or mRNA into targeted cells of living zebrafish embryos would be a great benefit. Thus, we attempted to manipulate the fate of single neurons in zebrafish embryos by introducing mRNA using our photoporation technique (Figure 4). Protein kinase A (PKA) is involved in the hedgehog signaling pathway in the central nervous system, and injection of a dominant-negative form of PKA mRNA (dn*PKA*) into single-cell embryos induces expression of the floor plate marker *spondin1b* (*spon1b*; previously known as *F-spondin*) in dorsal regions of the central nervous system [6]. We examined whether dn*PKA* mRNA could be introduced into single dorsal neurons of zebrafish embryos and looked for ectopic expression of *spon1b*. Because the zebrafish neural plate closes by the 14-somite stage (16 hpf) to form the neural rod, we injected dn*PKA* mRNA into the chorion cavity at 13–14 hpf and focused 400-nJ laser pulses on the presumptive dorsal neurons of the unclosed neural keel (Figure 4A). Immediately after irradiation, embryos were dechorionated, washed, and manipulated in fish water. At 24 hpf, *spon1b* transcripts were observed in the floor plate of embryos injected with either control *EGFP* mRNA or dn*PKA* mRNA, as expected (FP in Figure 4B, C, D). In addition, ectopic *spon1b* expression was detected in the dorsal neurons of embryos injected with dn*PKA* mRNA (arrowheads in Figure 4C, E). Introduction of a cocktail of biotin-dextran and dn*PKA* mRNA confirmed that the cells expressing ectopic *spon1b* were indeed photoporated (Figure 4D). These results clearly show that our photoporation technique can be applied for manipulation of gene function in single cells in higher vertebrates.

## Discussion

In this paper, we have shown for the first time that biomolecules can be introduced into individual cells of diverse live non-transgenic embryos using the presented femtosecond laser photoporation technique. This demonstration opens the opportunity to introduce various types of biomolecules, including DNA, RNA, MOs, and drugs, into single cells in a broad range of vertebrate embryos. Here we discuss the physical mechanism of our photoporation technique as well as its potential for application in a broad range of biological research settings.

The introduction of biomolecules into cells by photoporation is triggered by multiphoton absorption. We propose that the physical phenomena induced by multiphoton absorption in animal cells can be categorized as follows: (I) enhancement of membrane fluidity, (II) generation of a hole by laser ablation of the cell membrane, and (III) cell dispersion due to shockwave and cavitation bubbles at the laser focal spot. Enhanced membrane fluidity (I) seems to be due to weak heating of the cell membrane by laser irradiation. With increasing total irradiation energy, the accumulated heat leads to local disruption of the membrane. Furthermore, when the pulse energy is sufficiently high, the cell membrane can be disrupted by single-pulse laser ablation and overheating, which has been explained in terms of its photomechanical mechanism [7], [8]. These processes then lead to generation of a hole (II). The generation of a shockwave and cavitation bubbles occurs in response to an excessive single-pulse ablation, and has been explained in terms of optical breakdown [7]. When a low pulse energy (<1 nJ/pulse) and a high repetition rate (>10 MHz) are used, external molecules around the targeted cell are transported intracellularly as a result of phenomena I and II. On the other hand, our previous investigation [4] suggests that a high pulse energy (>100 nJ/pulse) and a low repetition rate (1 kHz) lead to the introduction of exogenous molecules mainly by phenomenon II. Notably, when a tissue cell layer is stimulated by a laser pulse with suitable energy to induce phenomenon II, exogenous molecules are delivered to single cells in the tissue (Figure S2A). If the laser pulse energy is excessively high (i.e., 800 nJ/pulse), however, phenomenon III is also induced, causing dispersal of cells surrounding of the laser focal point (Figure S2B). It is known that cell-cell adhesion in early embryonic tissues is relatively weak. The fact that the NIR-fs laser tended to disperse cells in early-stage zebrafish embryos (Figure 1D, E) can be explained by phenomenon III. Similar dispersion of cells was observed when NIR-fs laser pulses were applied to early-stage chick embryos (stage 14; Fig. 2D). However, the proportion of dispersed cells was not always proportional to the level of energy applied. This discrepancy could reflect individual differences in the geometrical relationship between sample and laser focal position, which leads to variability in the laser focus. If the laser spot is focused on the edge of the cells, cells may be dispersed with relatively lower energy. For similar reasons, success rates of delivery of molecules were not always proportional to the level of energy applied. The phenomena I, II, and III were induced at the laser focal spot even when the laser was focused on internal cells. Taking advantage of this observation, we were able to introduce plasmid DNA into single cells in the neural tube of chick embryos (Figure 2E–H).

This technique allowed us to introduce a great variety of biomolecules, including dextran, DNA, MOs, and RNA, into single cells of vertebrate embryos. The procedure was successful in a range of commonly used model organisms, including zebrafish, chick, and mouse. Furthermore, it was successful in a non-model organism, the shark, opening the opportunity to introduce biomolecules to single cells of a much broader range of organisms. Heretofore, local manipulation of gene function in animals generally required production of transgenic animals or the use of viral vectors, chemicals, or electroporation to transfect exogenous genes. Producing transgenic animals is time consuming and is only applicable in a limited range of organisms. Viral vectors, chemical transfection reagents, and electroporation cannot be used to target individual cells. Even the recent application of a continuous-wave infrared laser to target cells can only be applied to transgenic animals carrying a gene driven by a heat shock promoter. Our method will permit the functional genomic analysis of targeted cells in a potentially unlimited range of vertebrate embryos without the need to produce transgenic animals.

The introduction of DNA plasmids, MOs, and RNAs into vertebrate embryos has provided a general method for performing a variety of gain- or loss-of-function experiments (Table S1). Molecules can be introduced directly to the cell of one- or two-cell embryos, or they can be delivered to limited areas of later-stage embryos by electroporation or chemical or viral mediators. Although these methods are in widespread use, it has been difficult to limit the region that is transfected or to direct molecules to deep levels of embryos where molecules cannot be injected. Targeting deep-embryo sites poses a problem not only for electroporation or mediators but also for our photoporation method. For all these methods, molecules can be introduced more efficiently if the targeted regions lie next to or near a cavity with a clear lumen where molecules can be injected. Nonetheless, our photoporation technique provides an advantage in terms of the ability to restrict delivery to targeted cells.

It has also been difficult to apply current techniques to allow functional gene analysis in single cells of living embryos. In teleost fishes, introduction of MOs or mRNA can be carried out in one- or two-cell embryos by using glass capillaries, but delivering molecules to individual cells beyond the 32-cell stage has been challenging. Although focal electroporation has been achieved in zebrafish embryos in several cases [9], [10], [11], it came with the challenge of controlling the number of transfected cells. In chick and mouse embryos, although DNA plasmids have been introduced into targeted regions by electroporation or toxic mediators, targeted delivery has not yet been achieved at the single-cell level.

Using our photoporation technique method, we succeeded in introducing these biomolecules into targeted single cells without the use of toxic reagents. Moreover, this technique allowed us to introduce multiple biomolecules simultaneously in the same cells. Finally, we demonstrated that the introduction of mRNA into individual cells of live zebrafish embryos could manipulate the fate of those cells, again without the use of any toxic reagents. The development of new techniques for manipulation of cell fate and function by introducing multiple biomolecules into cells of living animals without the use of toxic mediators opens many opportunities for future biomedical research in areas such as gene therapy and induced pluripotent stem cell research, among others.

In conclusion, the NIR-fs laser approach for intracellular delivery of biomolecules should be adaptable to a wide range of non-transgenic organisms, obviating the time-consuming process of producing transgenic animals and allowing the manipulation of gene function in many non-model systems. We demonstrated the success of this method using a variety of biomolecules and a wide range of higher vertebrates. This technique thus holds great promise for improving the efficacy and accuracy of targeted transfection/delivery of biomolecules to individual cells in a broad range of research disciplines.

## Materials and Methods

### Ethics Statement

Experimental protocols involving animals were approved by Animal Studies Committees at NAIST (the permit numbers; 193 and 216).

### Experimental Equipment for NIR-fs Laser Photoporation

NIR-fs laser pulses from a regeneratively amplified Ti:sapphire femtosecond laser system (Spectra physics, Hurricane, 800 nm, 120 femtoseconds (fs), 1 mJ/pulse, 1 kHz) were focused on targetedepithelial cells or neurons in embryos through a 10× objective (Olympus, PlanN, 0.25 NA, 10.6 working distance) on an inverted microscope (Olympus, IX71) as shown in Figure 1A [12]. In this laser system, NIR-fs laser pulses generated by a Ti:sapphire femtosecond laser oscillator (Spectra-Physics, Mai Tai laser) were reduced to a repetition rate of 1 kHz and amplified by chirped-pulse regenerative amplification. Fifty repetitions of the amplified pulses were detected with a mechanical shutter (gate time 50 ms) and delivered to the sample. The laser pulse energy was tuned by a half-wavelength (λ/2) plate and dual polarizers. Laser pulses were collimated by dual convex lenses before the microscope, and the laser focal point was tuned to the plane of the image on the microscope. The laser pulse energy through the objective was measured by attaching a laser power meter (ORIEL, model AN/2) to the objective. The pulse energy after passing through the objective was approximately half that before the objective due to delivery loss. The spatial distribution of the laser intensity at the focal spot was estimated by examining the etching pattern on a carbon-doped polymer film, in which laser ablation is initiated by single-photon absorption. The diameter of the laser focal point was ∼5 µm. The threshold energy for cavitation bubble generation in water (*F_th_^W^*) was ∼50 nJ/pulse, which is estimated to be 250 mJ/cm^2^ as a unit of laser fluence. This is in rough agreement with the fluence reported as a threshold of optical breakdown [8], [13]. Namely, we performed the photoporation experiments with pulse energy ranging from 2×*F_th_^W^* (100 nJ) to 16×*F_th_^W^* (800 nJ). The *z* position was controlled by mechanically shifting the microscope stage from the image plane. The accuracy of the *z* position, based on the mechanical precision of the microscope stage, was less than 5 µm. Therefore, we can target cells in the *z* plane with this precision. The three-dimensional precision of targeting is sufficient for the experimenter to reliably target single cells, as the typical animal cell is between 10 and 100 µm (see Movie S1 and Movie S2). The process of laser-mediated biomolecule delivery was monitored by transmission images collected using a CCD camera (Ikegami, ICD-878). Total magnification of the images is 540×. The illuminating light source was a halogen lamp set above the microscope. The laser focal point in the image plane was adjusted using a joystick to position the microscope stage, after which the laser pulses were shot by opening the mechanical shutter. The accuracy of the *xy* position was less than 5 µm.

### Morpholinos, Dextran, Plasmids, and mRNA

Fluorescein-tagged standard control morpholino antisense oligonucleotides (control MOs, 5′-CCTCTTACCTCAGTTACAATTTATA-3′; Gene Tools) were dissolved in endotoxin-free water at a final concentration of 5 ng/nl. Fluorescein-conjugated 10,000 MW dextran and mini-ruby dextran (tetramethylrhodamine and biotin, 10,000 MW, lysine fixable) were purchased from Invitrogen. pCAGGS-EGFP [14] and pCAGGS-RFP [15] were dissolved in endotoxin-free water at a final concentration of 10 mg/ml. Capped mRNAs were transcribed from linearized pCS*EGFP* [7] and pCSdn*PKA* [6] using an *in vitro* transcription kit (MEGAscript SP6, Ambion).

### NIR-fs Laser Photoporation for Single Cells in Zebrafish Embryos

Wild-type *Danio rerio* (strains TL and Tü) were reared as described [16]. Embryos were mounted in the center of a depression on a glass slide with 3% methylcellulose solution under tricaine anesthesia (MS222, Sigma). For epithelial fibroblasts, 5 ng/nl FITC-MO, 5% FITC-dextran, or 5% mini-ruby dextran were injected into the cavity between the chorion and the embryo surface. Immediately after injection, the femtosecond laser was focused on the surface of a single epithelial cell. For neural cells, 400 ng/µl control EGFP mRNA [17] or dn*PKA* mRNA [6] in 2.5% FITC-dextran or in 2.5% miniruby-biotin-dextran was injected into the cavity between the chorion and the surface of an 8- to 10-somite embryo (13–14 hpf). Femtosecond laser pulses were focused on the presumptive dorsal neurons of the unclosed neural keel. After laser irradiation, eggs were manually dechorionated and washed in fish water [16]. At 24 hpf, some embryos were fixed in 4% paraformaldehyde/PBS for *in situ* hybridization.

### NIR-fs Laser Photoporation for Single Cells in Chick Embryos

HH stage 12–16 [18] chicken embryos were explanted with the aid of a ring of filter paper and cultured according to a modified version of the New culture method [5]. For epithelial cells, a solution of 5% mini-ruby dextran (Invitrogen) and/or 10 mg/ml pCAGGS-EGFP was injected between the vitelline membrane and the epiblast ectoderm. Femtosecond laser pulses were then focused on the surface of a single epithelial cell. For neural cells, a solution of 10 mg/ml pCAGGS-RFP was injected into the lumen of the neural tube, and a single cell was irradiated. Immediately after laser irradiation, embryos were transferred to a fresh albumin plate for New culture and incubated at 38°C.

### NIR-fs Laser Photoporation for Single Cells in Mouse Embryos

E9 mouse embryos with closed yolk sacs were precultured for 1–2 h in rat serum (Charles River Japan) as described [19], [20], and then transferred into Tyrode's solution (Sigma). Reichert's membrane was removed, and a solution of 5% mini-ruby dextran was injected into the lumen of the neural tube. A small slit was made in the yolk sac to allow femtosecond laser pulses to be focused on the surface of the neural tube. Immediately after laser irradiation, embryos were placed in a warm tube with 1 ml warm (37°C) rat serum per embryo. The tube was then gassed with 20% O_2_/5% CO_2_,/75% N_2_, sealed, and placed in a roller-tube mouse incubator at 37°C. Embryos were harvested after 24 h of culture and fixed in 4% paraformaldehyde/PBS.

### NIR-fs Laser Photoporation for Single Cells in Shark Embryos

A window ∼10 mm in diameter was opened on the surface of *Scyliorhinus canicula* eggs at stage 29. Embryos were removed from the eggs and transferred to saline solution for sharks [21]. A solution of 5% mini-ruby dextran was injected into the lumen of the neural tube, and the femtosecond laser was focused on a single neuron. Immediately after irradiation, embryos were returned to the eggs and incubated in a moist chamber at 16°C. Embryos were harvested after 3–7 days of culture and fixed in 4% paraformaldehyde/PBS.

### Immunohistochemistry

Immunohistochemical localization of proteins was performed as described [20]. The polyclonal antibody against RFP was purchased from MBL.

### Whole-Mount *In Situ* Hybridization and Biotin Labeling

Whole-mount *in situ* hybridization was performed as described [16]. The probe for *Danio rerio spondin 1b* (*spon1b*) was amplified by reverse transcription-polymerase chain reaction using primers derived from the published sequence (NM_131517). Selected stained embryos were processed for cryosectioning by dehydrating, incubating in 20% sucrose/PBS, and embedding in 7.5% gelatin/15% sucrose. Sections were ∼20–40 mm thick. For biotin labeling in combination with *in situ* hybridization, stained embryos were treated with 0.1 N glycine HCl (pH 2.2), washed five times in PBS, and incubated overnight with streptavidin–horseradish peroxidase (HRP; Vector) in PBS at 4°C. The specimens were washed five times in PBS at room temperature, then HRP activity was detected using 3,3′-diaminobenzidine in water with 0.01% hydrogen peroxide.

## Supporting Information

Figure S1 **Schematic examples indicating the difference between femtosecond laser–mediated introduction of DNA into a thin sample with high transparency (A) and a thick sample with low transparency (B).** (A) Conventional method for DNA introduction into cultured cells utilizing a Ti: Sapphire femtosecond laser oscillator with low pulse energy (<1 nJ/pulse) and a high repetition rate (>10 MHz). (B) Our method for introducing MO, DNA, or RNA into single cells in living mouse embryos utilizing the regeneratively amplified Ti: Sapphire laser system with high pulse energy (>100 nJ) and a low repetition rate (1 kHz).
(PDF)

Figure S2 **Multiphoton absorption leads to introduction of molecules to cells or cell dispersion.** (A) Generation of a shockwave and cavitation bubbles caused by an excessive single-pulse ablation leads to generation of a hole in the cell membrane and delivery of molecules to the cell through the hole. (B) Cell dispersion is induced when the pulse energy is particularly high.
(PDF)

Table S1 **Comparison of NIR-fs laser photoporation and other methods.**
(DOC)

Movie S1 **Typical example of targeting a single cell in a zebrafish embryo using a NIR-fs laser.** The arrow indicates the targeted cell at the surface of the zebrafish embryo. The black spot observed 33 ms after laser irradiation is due to local deformation of the cell, which leads an increase in light scattering.
(AVI)

Movie S2 **Demonstration of laser focal precision in the optical (** ***z*** **) axis.** A 200-nJ laser pulse was focused on cells in the neural tube of zebrafish embryos at the surface (arrow at “surface”) and at 15 µm from the surface in the direction of the *z* axis (arrow at “15 µm”).
(AVI)
