# Supplementary material for: Photoporation of Biomolecules into Single Cells in Living Vertebrate Embryos Induced by a Femtosecond Laser Amplifier
Source: PLoS One. 2011 Nov 16;6(11):e27677. doi: 10.1371/journal.pone.0027677 (PMC3218030; doi:10.1371/journal.pone.0027677)
Supplement: Figure S1 — Schematic examples indicating the difference between femtosecond laser–mediated introduction of DNA into a thin sample with high transparency (A) and a thick sample with low transparency (B). (A) Conventional method for DNA introduction into cultured cells utilizing a Ti: Sapphire femtosecond laser oscillator with low pulse energy (<1 nJ/pulse) and a high repetition rate (>10 MHz). (B) Our method for introducing MO, DNA, or RNA into single cells in living mouse embryos utilizing the regeneratively amplified Ti: Sapphire laser system with high pulse energy (>100 nJ) and a low repetition rate (1 kHz). (PDF) [file pone.0027677.s001.pdf]

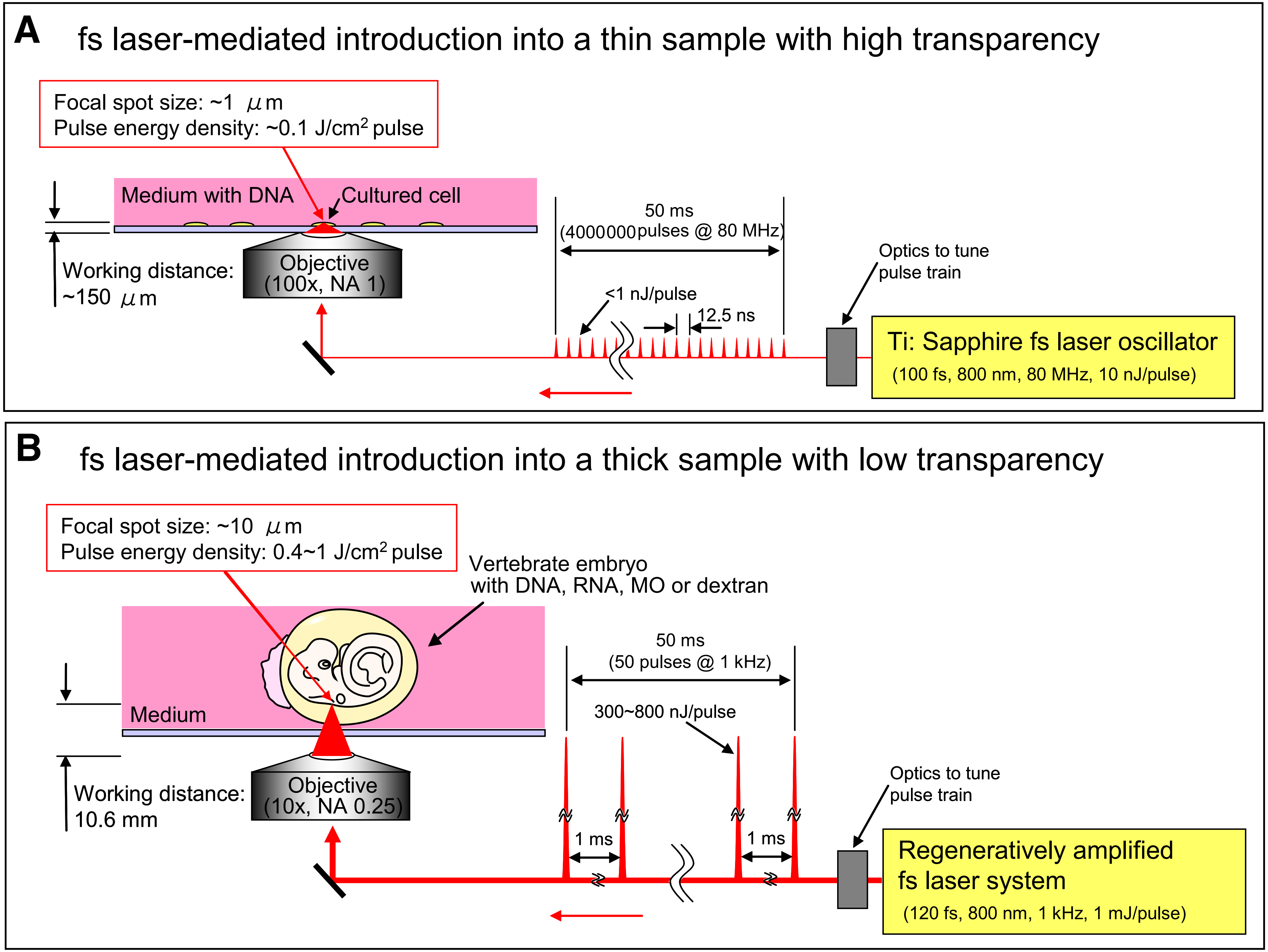

Fig. S1 Hosokawa et al.
