# Supplementary material for: Photoporation of Biomolecules into Single Cells in Living Vertebrate Embryos Induced by a Femtosecond Laser Amplifier
Source: PLoS One. 2011 Nov 16;6(11):e27677. doi: 10.1371/journal.pone.0027677 (PMC3218030; doi:10.1371/journal.pone.0027677)
Supplement: Figure S2 — Multiphoton absorption leads to introduction of molecules to cells or cell dispersion. (A) Generation of a shockwave and cavitation bubbles caused by an excessive single-pulse ablation leads to generation of a hole in the cell membrane and delivery of molecules to the cell through the hole. (B) Cell dispersion is induced when the pulse energy is particularly high. (PDF) [file pone.0027677.s002.pdf]

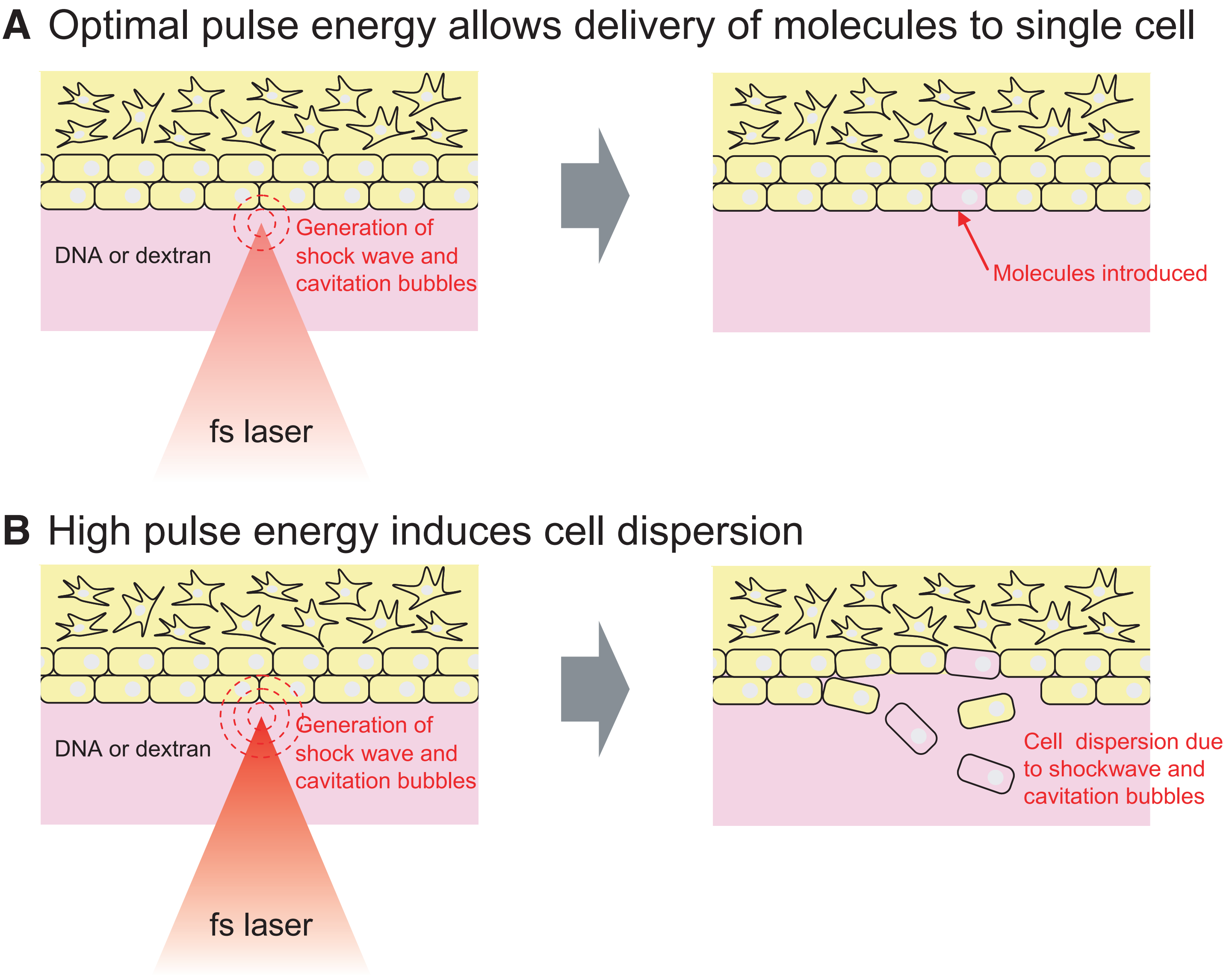

Fig. S2 Hosokawa et al.
