# Supplementary material for: Photoporation of Biomolecules into Single Cells in Living Vertebrate Embryos Induced by a Femtosecond Laser Amplifier
Source: PLoS One. 2011 Nov 16;6(11):e27677. doi: 10.1371/journal.pone.0027677 (PMC3218030; doi:10.1371/journal.pone.0027677)
Supplement: Table S1 — Comparison of NIR-fs laser photoporation and other methods. (DOC) [file pone.0027677.s003.doc]

**Table S1.** Comparison of NIR-fs photoporation and other methods

|  | NIR-fs photoporation | Electroporation | Lipofection | Viral vectors | IR-LEGO^[1]^ |
| --- | --- | --- | --- | --- | --- |
| Animal embryos method can be applied to | Wide range of non-transgenic animal embryos (mouse, chick, zebrafish, shark) | Wide range of non-transgenic animal embryos (mouse, chick, zebrafish) | Mouse, chick embryos | Animal embryos that can be infected by viruses (mouse, chick) | Transgenic animal embryos (worms, zebrafish) |
| Molecules method can be applied to | Wide range of exogenous molecules (DNAs, RNAs, morpholinos, dextrans) | Electrically charged exogenous molecules (DNAs, RNAs) | Electrically charged exogenous molecules (DNAs, RNAs) | Exogenous gene(s) in viral vector | Gene driven by heat shock promoter in transgenic animal embryos |
| Precision at single cell level | Highly efficient | Almost impossible (a few reports of success in zebrafish^[9], [10], [11]^) | Impossible | Impossible | Highly efficient |
| Toxicity | Nontoxic | Nontoxic | Low toxicity | Low toxicity | Nontoxic |
| Physical damage | Possible | Possible | None | None | None |
| Practically possible targeting of deep cells | Only if targeted regions lie near a cavity with a clear lumen where molecules can be injected | More efficient; when targeted regions lie near a cavity with a clear lumen where molecules can be injected | More efficient; when targeted regions lie near a cavity with a clear lumen where molecules can be injected | Highly efficient | Highly efficient |
